# Evidence on the magnitude of the economic, health and population effects of palm cooking oil consumption: an integrated modelling approach with Thailand as a case study

**DOI:** 10.1186/s12963-019-0191-y

**Published:** 2019-08-16

**Authors:** Marcus R. Keogh-Brown, Henning Tarp Jensen, Sanjay Basu, Wichai Aekplakorn, Soledad Cuevas, Alan D. Dangour, Shabbir H. Gheewala, Rosemary Green, Edward JM Joy, Nipa Rojroongwasinkul, Nalitra Thaiprasert, Bhavani Shankar, Richard D. Smith

**Affiliations:** 1 Department of Global Health and Development, Faculty of Public Health and Policy, London School of Hygiene and Tropical Medicine, 15-17 Tavistock Place, London, WC1H 9SH UK; 2 University of Copenhagen, Copenhagen, Denmark; 3 Stanford University, Stanford, USA; 4 Ramathibodi Hospital, Mahidol University, Nakhon Pathom, Thailand; 5 Department of Population Health, Faculty of Epidemiology and Population Health, LSHTM Keppel Street, London, WC1E 7HT UK; 6 King Mongkut’s University of Technology Thonburi (KMUTT), Bangkok, Thailand; 7 Institute of Nutrition, Nakhon Pathom, Thailand; 8 Chiang Mai University University, Chiang Mai, Thailand; 9 SOAS University of London, London, UK; 10 College of Medicine and Health, Exeter University, Exeter, UK

**Keywords:** Palm oil, Diet, Nutrition, Disease burden, CGE, Macroeconomic

## Abstract

**Background:**

Palm oil’s high yields, consequent low cost and highly versatile properties as a cooking oil and food ingredient have resulted in its thorough infiltration of the food sector in some countries. Longitudinal studies have associated palm oil’s high saturated fatty acid content with non-communicable disease, but neither the economic or disease burdens have been assessed previously.

**Methods:**

This novel palm oil-focussed disease burden assessment employs a fully integrated health, macroeconomic and demographic Computable General Equilibrium Model for Thailand with nine regional (urban/rural) households. Nutritional changes from food consumption are endogenously translated into health (myocardial infarction (MI) and stroke) and population outcomes and are fed back into the macroeconomic model as health and caregiver-related productive labour supply effects and healthcare costs to generate holistic 2016–2035 burden estimates. Model scenarios mirror the replacement of palm cooking oil with other dietary oils and are compared with simulated total Thai health and macroeconomic burdens for MI and stroke.

**Results:**

Replacing consumption of palm cooking oil with other dietary oils could reduce MI/stroke incident cases by 8280/2639 and cumulative deaths by 4683/894 over 20 years, removing approximately 0.5% of the total Thai burden of MI/stroke. This palm cooking oil replacement would reduce consumption shares of saturated/monounsaturated fatty acids in Thai household consumption by 6.5%/3% and increase polyunsaturated fatty acid consumption shares by 14%, yielding a 1.74% decrease in the population-wide total-to-HDL cholesterol ratio after 20 years. The macroeconomic burden that would be removed is US$308mn, approximately 0.44% of the total burden of MI/stroke on Thailand’s economy or 0.003% of cumulative 20-year GDP. Bangkok and Central region households benefit most from removal of disease burdens.

**Conclusions:**

Simulations indicate that consumption of palm cooking oil, rather than other dietary oils, imposes a negative health burden (MI and stroke) and associated economic burden on a high consuming country, such as Thailand. Integrated sectoral model frameworks to assess these burdens are possible, and burden estimates from our simulated direct replacement of palm cooking oil indicate that using these frameworks both for broader analyses of dietary palm oil use and total burden analyses of other diseases may also be beneficial.

**Electronic supplementary material:**

The online version of this article (10.1186/s12963-019-0191-y) contains supplementary material, which is available to authorized users.

## Background

Vegetable oils are a major component of food systems and an important economic commodity. Since 1980, the global use of vegetable oils has increased by a factor of 4.5. In particular, due to its proportionally high yields [1], palm oil consumption has grown tenfold since 1980, exceeding that of soybean oil and constitutes around one-third of global vegetable oil consumption (57 million tonnes) [2], three-quarters of which is used for food. Palm oil’s pervasive use throughout food systems derives from its high yields and consequent low price amongst dietary oils but also from its versatility: its stability makes it ideal for frying purposes and its texture and oxidisability make it useful as a processed food ingredient and give it preservative properties [3]. However, palm oil contains a very high ratio of saturated fatty acids (SFA) to polyunsaturated fatty acids (PUFA) and monounsaturated fatty acids (MUFA) and increased consumption of SFAs relative to MUFA and PUFA has been linked to increases in serum cholesterol [4, 5] and, in turn, to increased incidence and mortality of myocardial infarction (MI) and stroke [6] and, more broadly, to increased rates of death from cardiovascular disease [7]. These diverse properties therefore place palm oil at the intersection between economic, health and nutritional policy interests. Recent rapid increases in production and consumption of palm oil are expected to continue, potentially increasing associated burdens of non-communicable disease, and this has led to recommendations that future long-term palm oil strategies should incorporate both economic interests and population health benefits [8]. However, the size of palm oil’s contribution to disease burdens and its economic impact are largely unknown and no macroeconomic and health burden estimates exist to quantify combined economic, disease burden and population impacts alongside other policy perspectives. This computable general equilibrium (CGE) analysis aims to fill this gap by providing an integrated quantification of the combined macroeconomic, disease and population burden of palm cooking oil consumption in a major palm oil consuming country context, Thailand.

### Palm oil’s effect on health

Palm oil is made up from 40 to 50% SFAs [9], and has the highest saturated fat content of vegetable oils, commonly used in cooking and food production, except for coconut oil [10]. Increased saturated fat consumption is thought to increase serum cholesterol [4, 5] and increase rates of death from cardiovascular disease [7], but the relationship between dietary oils, saturated fat and health is complex. Historical longitudinal studies have shown that *replacement* of saturated fats with polyunsaturated or monounsaturated fats produces a substantive and statistically significant reduction in cardiovascular risk from MI and stroke [11–13]. While some recent evidence has questioned the relationship between SFA intake and clinical health outcomes [14, 15], other compelling evidence supports the replacement of saturated fat with unsaturated fats and affirms the link between changes in palm oil consumption, serum cholesterol and incidence and mortality of MI and stroke [16, 17]. It has been suggested that palmitic acid (the saturated fatty acid which makes up 35–45% of palm oil fatty acids) has little effect on the lipid profile of individuals [18], but this stands in contrast to other evidence that palm oil consumption results in higher LDL cholesterol than do vegetable oils low in saturated fat [6, 9]. There is also strong evidence from large-scale longitudinal studies, conducted over 24–28 years, showing that replacement of saturated lauric, myristic and stearic acid yields a 6–8% reduced risk of coronary heart disease, while replacement of palmitic acids yields a larger 10–12% reduction in risk [19]. Current long-term results therefore present convincing evidence for the health benefits of replacing saturated fats from palm oil with poly- and monounsaturated fats and support the methods presented in Mensink et al. [4]. These methods are particularly applicable in analyses of the type presented here which focus on the pure nutritional effect of consuming palm cooking oil relative to other dietary oils and they are therefore the basis for the health modelling underlying our disease burden estimations.

### The usefulness of palm oil for food

Palm oil’s properties relative to alternative vegetable oils have been summarised elsewhere [3] and, in addition to wide availability and low cost, include suitability and superiority both as a frying oil and for a wide range of processed food applications. It is low in harmful trans fatty acids but its stability and oxidative properties result in a longer shelf life for foods that contain it. However, many of the properties of palm oil which are beneficial for use in food are derived from its very high saturated fat content. Replacing palm oil in food processing applications such as bakery and confectionery products is costly and complex in terms of practical implementation. However, replacement of palm oil in applications which involve small amounts of fat and replacement of palm oil with other liquid oils for domestic frying purposes is more feasible [20]. Therefore, focussing on palm cooking oil and estimating the nutritional and disease burden effects which result from its consumption by households is preferable for our modelling of economic commodities and nutritional impacts.

### Country context

In many countries, palm oil is the predominant cooking oil and the cheapest oil for food use. In countries with a comparative advantage in palm oil production, yields per hectare of land can be ten times that of soybean oil [1]. In Thailand, the world’s third largest producer [21] and the context for this study, the production and availability of palm oil in the domestic market is high constituting 36% of the vegetable oil supply at 7.5 g/capita/day [22], and it is used in a wide range of foods. Since Thailand’s future plans for palm oil sector development include production for domestic use, with a focus on self-sufficiency and food security [23], and proposals to expand future palm plantation areas by 40%, increase oil extraction rates by 10% and encourage growth in consumption by 3% p.a. [21], it is an ideal context to conduct this assessment of macroeconomic and health burdens of palm cooking oil consumption.

### What is known about the economic and health burdens of palm oil?

Few studies have quantified the specific burden on health and health costs of palm oil consumption. Health studies have used logistic regressions to show that switching from palm to soybean consumption in Costa-Rica would reduce the burden of MI [24], while linear panel regressions have linked increased palm oil consumption to higher ischemic heart disease mortality rates in developing countries [25]. An economic-epidemiologic model has also been applied to simulate the effects of food taxation of palm oil consumption in India [6]. Furthermore, the World Health Organisation has referred to the link between palmitic acid consumption and cardiovascular disease as ‘convincing’ [26], but the disease burden, attributable to palm oil, remains to be quantified.

Economic studies exclude health but have linked palm oil production to reduced poverty and higher gross regional product [27], and to positive effects on smallholder income but also to increased inequality in palm oil farmer contracts [28–30]. In addition, oil palm cultivation has been shown to have significant positive effects on farmers’ livelihoods by increasing farm households’ consumption, and by creating efficiencies in farming activities and secondary gains from the time saved on farm labour [31].

Modelling studies of palm oil include partial equilibrium models which analyse how shifts in oil consumption patterns affect palm oil consumption in high consuming countries [32] and also include both global and single-country general equilibrium models which analyse the potential benefits of acceleration in oil palm yield growth in Indonesia and Malaysia [33] and capture the benefits of increased export taxes and higher world market prices for palm oil on household welfare in Indonesia [34]. Other multi-faceted modelling studies have employed a systems dynamic modelling approach to capture environmental, economic, social and human welfare perspectives of palm oil policies [35] and another study examined health, nutrition and economic aspects of oil palm adoption by farmers using survey data and econometric models [36]. While the latter study analysed household living standards, nutrition, food and non-food expenditures, calorie consumption and dietary quality, none of the aforementioned studies attempts to assess the health and macroeconomic burdens attributable to palm oil.

Broadening the scope beyond palm oil-specific studies, economy-wide simulation studies of health policy scenarios have been applied to assess adoption of healthy diet regulations [37], infectious disease pandemics [38] and environmental issues with health impacts or co-benefits [39, 40]. Some CGE studies have used satellite epidemiological models for infectious diseases [41–43] or nutrition measured through application of fixed caloric food coefficients (but do not fully integrate the clinical health feedbacks to the economy) [44, 45]. Also, one study linked satellite models to a CGE model to form a comprehensive economic, environmental, nutritional and clinical health model framework for the EU [46] but this model does not fully integrate a nutrient-related serum cholesterol biomarker health pathway as employed in this study. Therefore, whilst there are studies which demonstrate the usefulness of the CGE approach and whilst methodologies are moving towards full integration of health in CGE models, including an integrated CGE disease burden study for infectious disease [47], there are no disease burden studies which use fully integrated health and macroeconomic models to assess non-communicable disease, or attempt to measure health and macroeconomic burdens for palm oil consumption.

## Methods

In order to assess the health-related burden of palm cooking oil consumption across the whole economy, we employ a sectoral macroeconomic approach, computable general equilibrium (CGE) modelling. An important methodological development for our disease burden assessment is the full integration of a health modelling and demographic modelling component within the CGE model which together capture the cyclical feedback effects between the macro-economy, household consumption, health and the population. A detailed specification of the model and datasets is provided in [48], but a summary of its value and implementation is provided here.

### Modelling health-related disease burdens

World Health Organisation (WHO) guidance has highlighted the heterogeneous and disparate nature of disease burden estimation methods across clinical, epidemiological and economic perspectives. Even within health economics, the benefits and shortcomings of different approaches is elucidated: capturing health-related productive labour supply effects and macroeconomic impacts of demographic change is highlighted as important, as is capturing income effects at the household level. However, whilst economic, epidemiological and population indicators are important, WHO guidance also highlights the importance of distinguishing market and non-market costs and caregiver time losses [49]. Satisfying so many apparently exclusive goals would appear to require multiple tools and techniques but, as we shall show, it is possible for a single tool to simultaneously address them. The palm oil-focussed disease burden assessment method, employed in this study, addresses many of these apparently conflicting goals and perspectives by fully integrating models of the macro-economy, health and demographics in a single holistic model framework. The model captures macroeconomic productive labour supply effects, from patients and caregivers, and health system costs (economic disease burden), but also produces separate health indicators relating to cases, deaths and caregiver losses (health-related disease burden), and population level indicators, and disaggregates all outputs at the household level. Furthermore, by fully integrating the models, rather than running them in isolation, the disease burden estimates also account for interactions and feedback effects between health, the macro-economy and the population. The omission of feedback effects has been previously highlighted as a weakness in macroeconomic modelling studies of pandemic influenza disease burden simulations [50], and although adjustments in the design of simulation scenarios to account for feedback have been attempted elsewhere [38, 51], this study constitutes the first disease burden focussed study, of a non-communicable disease, based on a fully integrated macroeconomic, health and population model.

Furthermore, since this is the first non-communicable disease burden estimation of its kind, context is provided by performing additional analyses which use the same model framework to estimate the total myocardial infarction (MI) and stroke health burden in the same country context. Health modelling in the palm oil focussed simulations also relates to MI and stroke. The country selected for this analysis is Thailand, a major producer and consumer of palm oil which not only focuses on domestic use of the palm oil it produces but also regulates the formulation of its biofuels to ensure priority of palm oil supply for food use. The importance of palm oil to Thai diets, and Thailand in general, makes it the ideal context for a health-focussed disease burden assessment.

### Macroeconomic modelling component

The CGE model, employed in this study, is a sectoral mathematical model of the whole economy, which extends the fully documented IFPRI standard model [52]. The model captures the cost minimising and profit maximising behaviour of producers as well as consumption and saving behaviour of households and government, taxation mechanisms and the use of labour, capital and other factors in order to produce goods and services for investment or consumption. It also includes trade across international borders. The specification of production behaviour enables health-related labour changes, across all sectors, to be captured and valued at a dynamic wage level which adjusts, endogenously, according to economic growth, and to expansion or contraction of sectors in response to policy impacts over time. The relevance of the dynamic, multi-sector, household level modelling approach, and the CGE methodology itself, to capture productive labour supply impacts and estimate disease burdens, has been highlighted previously [49].

The CGE modelling process involves finding a benchmark solution (representing the current economy), and then simulating shocks, such as the removal of disease burdens to produce a new solution for comparison with the benchmark in order to estimate the economic impact of the simulated policy/event. This method has been used previously with satellite health models [53].

Our CGE model is calibrated from a 2007 Thailand Social Accounting Matrix (SAM) data set [54] and the original 260 sectors were aggregated into 49 commodities including 20 primary agricultural and food sectors. The model distinguishes nine households by region and urban/rural classification using data from the 2011 Household Socio-Economic Survey [55]. The CGE model was further extended to include an Almost Ideal Demand System (AIDS) specification of private demand for each of our nine regional households, based on price and income elasticities derived from the literature [56] with supplementary data from a recent Thai-specific food demand system study [57] and additional (non-Thai) edible oil cross-price elasticities [58, 59], to provide detail for primary agricultural and edible oil sectors, documentation of which is available elsewhere [48].

### Model integration

When modelling disease burdens from multiple perspectives, interaction and feedback effects between those perspectives may be important. For example, the removal of disease burdens through changes in food consumption may produce health and demographic effects which feed into labour supply and production affecting all sectors and creating ripple effects throughout the economy for income, savings and consumption. However, since these ripple effects cannot be known in advance and since consumption patterns will continue to affect health and feedback effects over time, these impacts can only be fully captured if economic, health and population impacts are calculated, endogenously, as part of an integrated simulation process. Whilst our model can be used to employ policy instruments and/or for broader non-health applications, we limit our integrated model application, in this case, to simulate the macroeconomic, health and demographic burden of changes in nutritional intakes of households related to their consumption of palm oil to mirror the replacement of palm cooking oil with consumption of ‘other dietary oils’. By this means, we isolate the disease-burden impact of palm oil consumption.

**Fig. 1:**
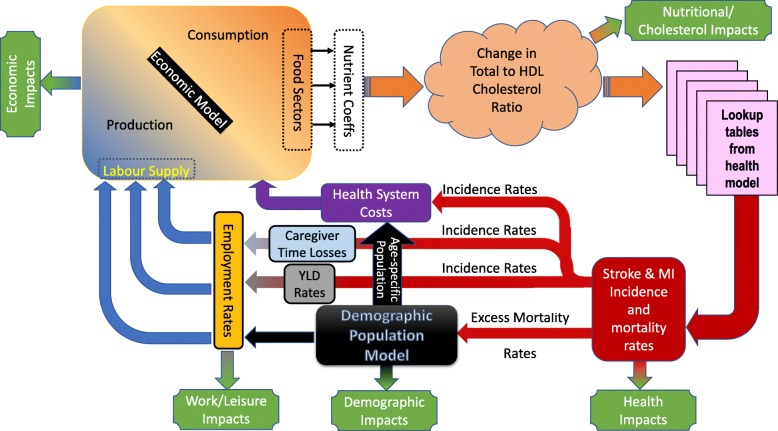
Model diagram. Diagram of integrated model framework

The model integration we employ is illustrated in Fig. 1. The central feature of the non-economic part of the model is the inclusion of nutritional weights for food sectors, a demographic model and polynomial approximations to age- and gender-specific lookup tables derived from a micro-simulation health model to translate changes in nutritional exposure to clinical health outcomes. As illustrated in Fig. 1, changes in household-specific demands for food commodities (including palm cooking oil) are multiplied by nutritional weights in order to calculate the changes in the nutritional profiles of household consumption (particularly those relating to SFA, PUFA and MUFA). The changes in the ratios of fatty acids consumed are translated to changes in total-to-HDL cholesterol ratios using parameters from the literature [4], and the changes in cholesterol biomarker values are, subsequently, used to compute changes in clinical health based on age- and gender-specific polynomials linking cholesterol biomarker build-up to MI- and stroke-related incidence and excess mortality rates. The health model, adapted for this purpose, has been previously published [6, 48]. Furthermore, the clinical health lookup tables, underlying the age- and gender-specific clinical health polynomials, were derived from a simulated cohort of 10,000 individuals for whom the mean and standard deviation of total/HDL cholesterol ratio were taken from the Thai National Health Examination Survey. Simulated incidence numbers, and years lived with disability (YLD) weights from the literature [60], are used to estimate morbidity impacts and incidence rates. These are combined with Thailand-specific average time loss estimates [61] and used to estimate caregiver leisure and worktime losses. Incidence rates, together with baseline unit costs for MI [62] and stroke [63], are used to calculate healthcare costs. Finally, morbidity, mortality and caregiver time losses are adjusted, using Thailand-specific workforce participation rates [64], and applied in the demographic module to estimate changes in productive labour supply, which are used as feedback effects into the macroeconomic model. More information on the sub-models of the integrated framework is provided in (Additional file 1: Appendix A) and full documentation is available elsewhere [48].

### Model scenario

The method employed in order to assess the health-related burdens associated with palm cooking oil consumption in Thailand is a nutrient-matching scenario which isolates the additional health, macroeconomic and population impacts that result from consuming "palm cooking oil" rather than “other dietary oils” (but assumes that there were no cost or supply constraints to allow a direct replacement of palm with other dietary oils). In practice, the nutrient-matching scenario involves changing the nutrient coefficients for palm cooking oil to match those of other types of edible oils. This approach does not use an economic policy instrument to encourage replacement of palm cooking oil with another edible oil, but mirrors a ‘magic bullet’ whereby the differences in nutritional properties of palm cooking oil, relative to other edible oils, are instantly aligned without policy or intervention and where no change in price or tax or other incentives are imposed in order to accomplish the change, thus isolating the ‘pure diet’ effect. The CGE model does adjust to capture the impact of this shock: household food consumption quantities will initially remain the same, but the changed nutritional exposures will feed into the health model and result in changes in total-to-HDL cholesterol ratios, and health outcomes will filter through into the demographic model, in the form of patient and caregiver-related productive labour supply changes as shown in Fig. 1, which, together with changes in healthcare expenditure, will feed back into government and household budgets in the macroeconomic model. The model will, subsequently, find a new equilibrium which reflects the removal of the additional burden of palm cooking oil on health and the economy, relative to consumption of other edible oils. This burden estimate represents the additional nutritional health and economic burden of household consumption of palm cooking oil in Thailand over other oils but does not prescribe a policy in order to remove it. Simulations are run for 20 years (2016 to 2035) and exposure changes are applied from the first year of simulation. As such, our burden estimates do not capture impacts from palm oil consumption in previous years, which may be realised in the current time period, but only include those impacts which result (and accumulate) from changes in palm oil consumption during the 20-year time horizon of our simulations. The 20-year timescale was selected as sufficiently short to maintain reliability of estimation whilst being long enough to allow accumulation of workforce effects. We also employ an additional simulation where palm oil nutritional coefficients are unchanged but where the total excess health burden of MI and stroke in Thailand (including patient and caregiver-related productive labour supply changes and changes in healthcare expenditure) are removed from the counterfactual. The removal of excess health burdens was possible since our clinical health lookup tables translate changes in total-to-HDL cholesterol ratio into changes in incidence and excess (rather than total) mortality rates for MI and stroke. The endogenous simulation of wage rates and healthcare unit costs also ensures that the calculation of total health benefits of illness elimination (the economic disease burden) is model consistent and takes account of future healthcare unit costs and labour market feedback effects (see Additional file 1: Appendix A for details about modelling of healthcare unit costs). This estimate of the “total” disease burden provides a methodologically consistent comparator for our nutrient-matching scenario results. Finally, we explore the impact of parameter uncertainty, related to the key serum cholesterol biomarker health pathway parameters from Mensink et al. [4], by utilising a previously published Monte Carlo simulation methodology [65, 66] and adapting it for use in our Thailand CGE model in order to produce sensitivity analyses of our results. The procedure was based on 250 sets of independent draws of SFA, MUFA and PUFA parameters from normal distributions, with prior means and standard deviations derived from Mensink et al. The number of independent draws ensures that average point estimates, for all outcome measures, have a precision of < 3.5 percentage points at the 95% confidence level.

## Results

Economic and health disease burden estimates from our simulations are presented in Table 1. All of the economic burden estimates in the table account for changes in productive labour supply due to morbidity, mortality or loss of caregiver time amongst working age employed people, and for healthcare costs. The health indicators which drive these burdens, including incidence and deaths, are also reported.

**Table 1:** Economic and clinical health burden results

	Total MI/stroke burdens	MI/stroke burdens from palm cooking oil (95% CI)	% of total (95% CI)
Economic burden (mn USD)			
Cumulative real GDP	69,488	308 (149, 467)	0.44% (0.22%, 0.67%)
Clinical health burdens (1000′)			
Cumulative life-years lost	15,264	49.9 (24.2, 75.7)	0.33% (0.16%, 0.50%)
Cummulative incident cases			
MI	1694	− 8.3 (− 12.6, − 4.0)	0.49% (0.24%, 0.74%)
Stroke	2058	− 2.6 (− 4.0, − 1.3)	0.13% (0.06%, 0.19%)
Cummulative deaths			
MI	1023	− 4.7 (− 7.1, − 2.3)	0.46% (0.22%, 0.69%)
Stroke	880	− 0.9 (− 1.4, − 0.4)	0.10% (0.05%, 0.15%)

### Total disease burden results

The first column of Table 1 illustrates the impact of removing the total disease burden attributable to stroke and MI in Thailand. This burden consists of 1.7 million MI cases and 2.1 million stroke cases resulting in an estimated 1 million MI deaths and 880,000 stroke deaths in Thailand over our 20-year time horizon. The cumulative economic impact of the total economic burden of MI and stroke in Thailand, estimated in real gross domestic product (GDP) terms, is approximately US$69bn (Table 1), while dynamic population impacts range from approximately 71,000 persons in 2016 up to 1.5 million persons in 2035 (not shown). The trajectory is approximately linear. Removing this burden entirely is not achievable as it would represent the complete removal of MI and stroke in Thailand. However, estimating this burden, using our integrated framework, provides a helpful comparator for our palm cooking oil disease burden simulation.

### Nutrient matching results

**Fig. 2:**
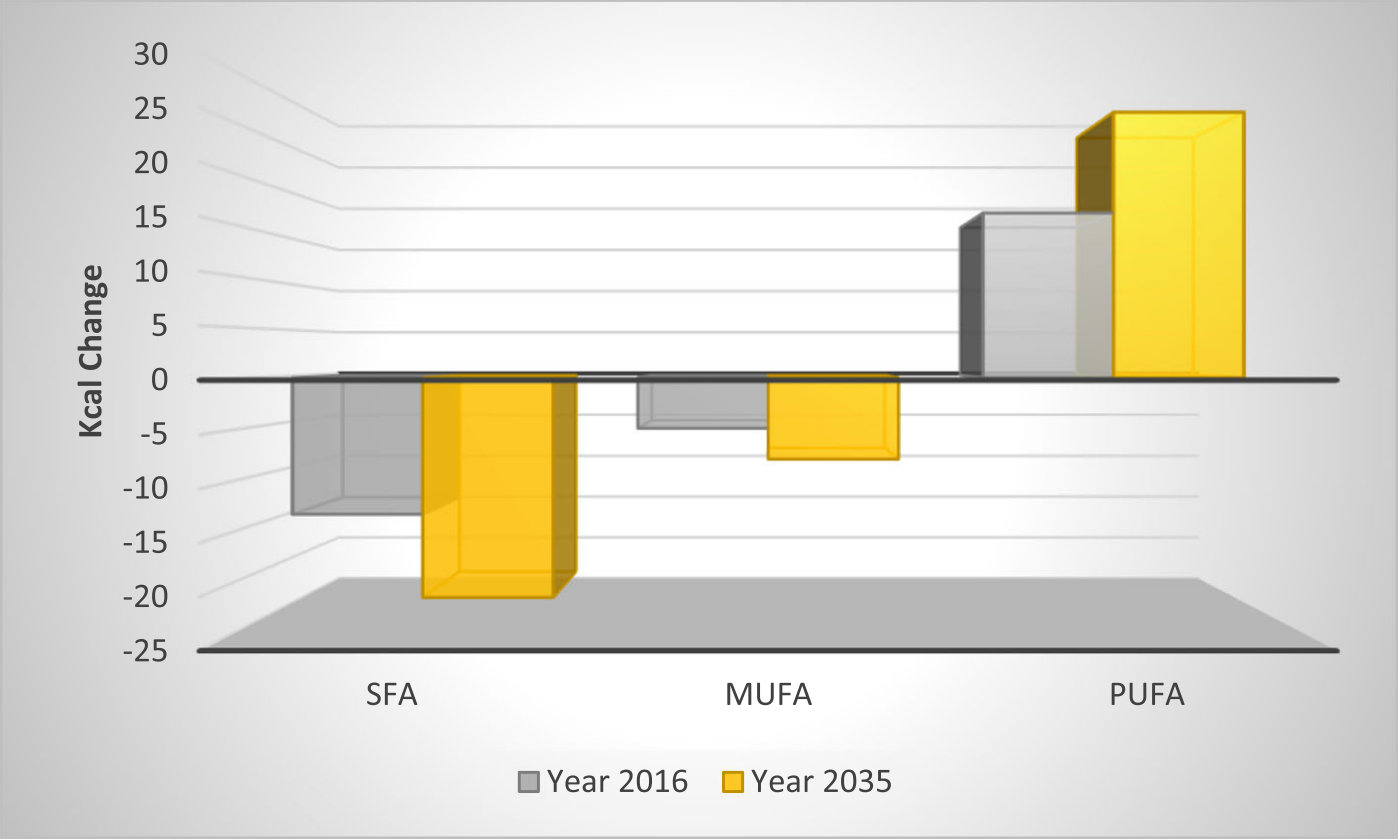
Aggregate fatty acid intakes (Kcal). Aggregate energy and fatty acid consumption changes

The nutrient matching simulation results illustrate the additional burden of household consumption of palm cooking oil over and above the burden that would be imposed by consumption of ‘other’ edible oils. The central estimates of our confidence intervals indicate that health burdens account for less than 0.5% of total burden values and suggest that incidence of MI and stroke would be expected to decline by 8280 and 2639 cases, respectively, and cumulative MI and stroke deaths by 4683 and 894, respectively, if an aggregate of other oils could be introduced, in a costless way, to replace household consumption of palm cooking oil over our 20-year time horizon (Table 1). Vegetable oils contribute, on average, approximately 14% of Thai daily SFA energy intake, with variation (from 11 to 19%) by region and urban/rural area (not shown). The changes in disease burdens are driven by the increased consumption share of PUFA (11.68% to 13.67% over the simulation period), slightly reduced consumption share of MUFA (− 3.04% to − 3.00% over the simulation period) and reduced consumption share for SFA (− 6.84% to − 6.49% over the simulation period). A further decomposition of changes in fatty acid consumption in terms of Kcal changes is shown in Fig. 2. These changes in exposure result in a linear population-wide decline in average cumulative total-to-HDL cholesterol ratio from 0.004 to 0.080 over the simulation period (a decline of between 0.09 and 1.73% in the total-to-HDL cholesterol ratio). The size of the economic disease burden is estimated to be US$308mn, just under half of 1% of the total economic burden and 0.003% of Thailand’s expected cumulative GDP over our time horizon (not shown).

**Fig. 3:**
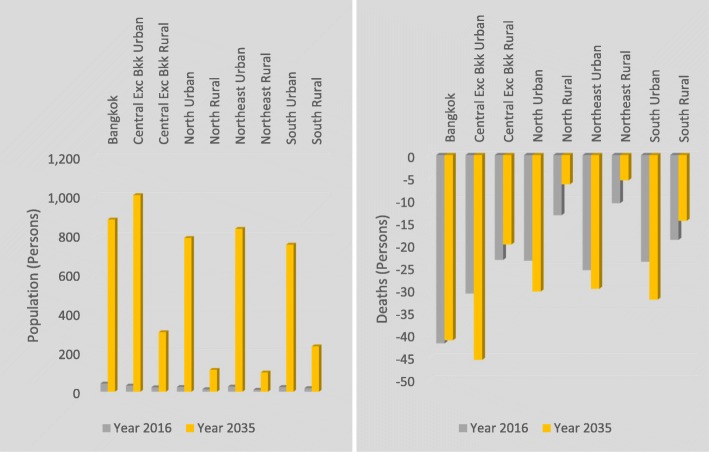
Changes in household population and deaths (persons). Disease burden impact on population and deaths by household

**Fig. 4:**
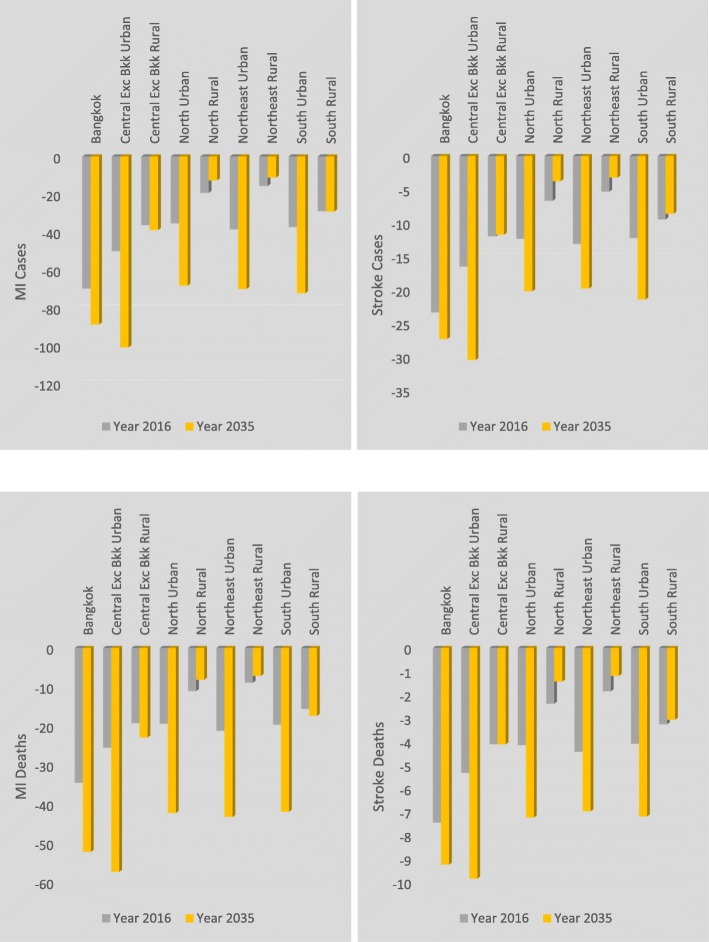
MI and stroke disease burden by household. Disease burden, cases and deaths from MI and stroke by household

**Fig. 5:**
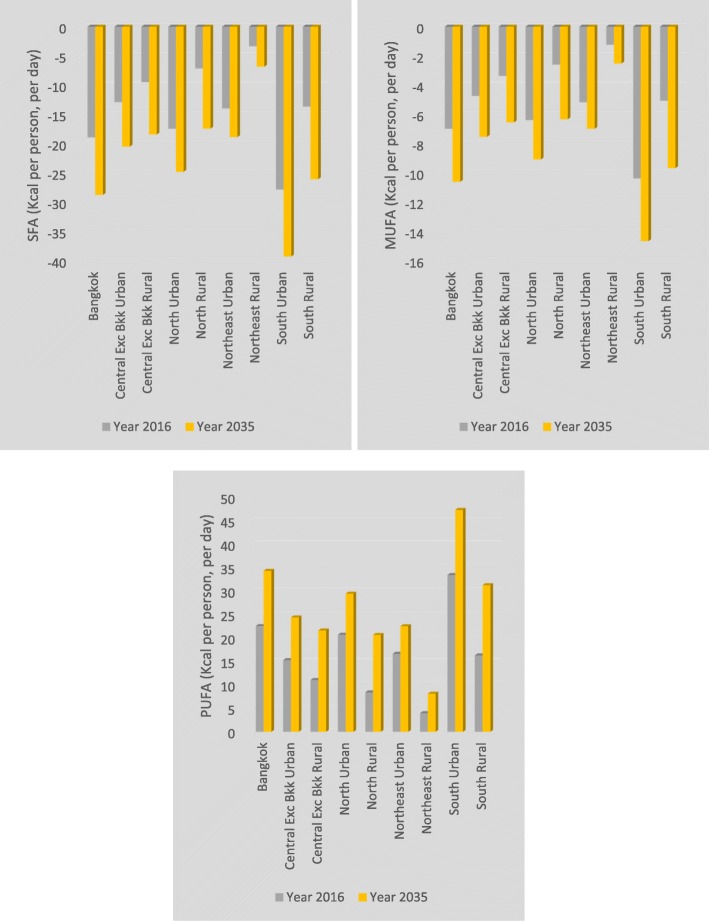
Household decomposition of fatty acid consumption. Changes in daily energy intake from fatty acid consumption by household

Consumption patterns differ between household types and economic, health and population outcomes reflect this. A decomposition of household impacts is provided in Figs. 3, 4 and 5. In general, a transition away from household consumption of palm cooking oil towards other oils would generate a larger reduction in disease burden (with accompanying health benefits and population increase) in urban than in rural areas, and population impacts accumulate over time showing much larger population gains in 2035 than in 2016. The Bangkok and Central regions experience the largest benefits from the removal of disease burdens. Southern, Northeast and Northern regions exhibit similar reductions in disease burdens, although comparing these three regions amongst themselves, the Southern region reductions in disease burden are marginally larger for urban areas and more than twice as large for rural areas. These differences by location and urban/rural categorisation reflect the higher levels of palm cooking oil consumption in urban than rural areas and primarily reflect differences in consumption and population size by region. Consumption of palm cooking oil in the rural South region, where the majority of palm cooking oil is produced, is greater than in central and northern rural regions and this is reflected in the disease burden estimates. Tables to further illustrate the nutrient matching effects with household disaggregation are shown in (Additional file 1: Appendix B).

**Fig. 6:**
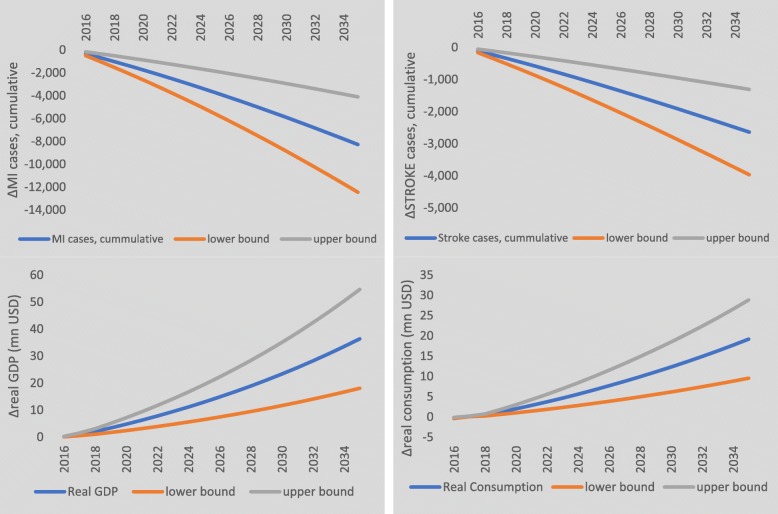
Dynamic health and economic burden effects. Dynamic MI, stroke, GDP and real consumption effects with confidence intervals

Figure 6 illustrates the dynamic trajectory of the economic and health burdens. By consuming other oils in place of palm cooking oil, the estimated number of MI incident cases averted increases slowly over time from approximately 330 to 490 per year in order to reach the cumulative total of 8280. Stroke cases averted similarly increase from 111 to 146 per year totalling 2639. The corresponding annual real GDP impacts increase in magnitude from US$160,000 in 2016 to US$36.4mn in 2035. Real (private) consumption, which encompasses all Thai consumption items including agricultural and processed foods, manufactures and all types of services including medical services, is also shown. After an initial decrease in the first year, real consumption rises annually from US$140,000 in 2017 to US$19.2m in 2035.

**Fig. 7:**
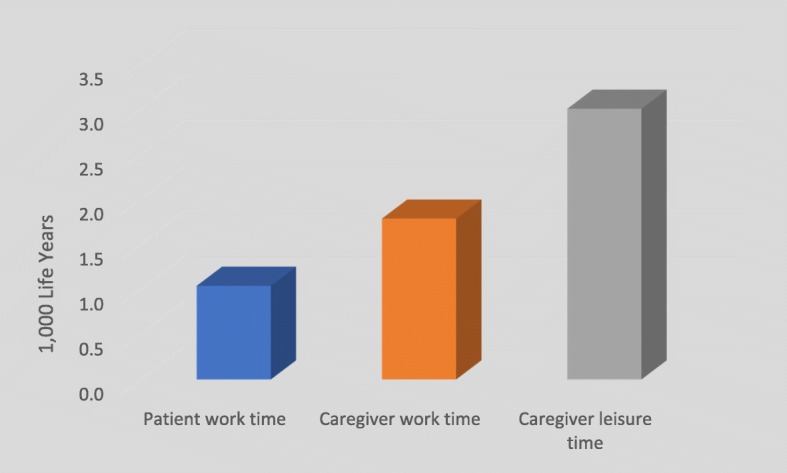
Patient and caregiver time losses. Patient and caregiver work and caregiver leisure time losses

A decomposition of the cumulative patient and caregiver time losses is also provided in Fig. 7. Caregiver work-time lost (1785 person-years) is greater than patient work-time lost (1037 person-years). Leisure time lost by caregivers does not contribute to the economic burden but amounts to 3001 person-years over our 20-year time horizon.

### Sensitivity analysis

The sensitivity analyses of our nutrition matching simulation results, to changes in key health model parameters, are shown in Table 1 and Fig. 7. Table 1 shows that the upper/lower cumulative burdens differ from the central estimates by ± 50% giving a range for the clinical health impacts of between 24 and 76 thousand cumulative life-years lost and a range for the economic impact of US$149mn to US$467mn. The dynamic results are shown in Fig. 6 and, similar to the cumulative burdens, the upper/lower values of the confidence intervals vary from the central estimates by approximately ± 50% year on year. These sensitivity results illustrate that, whilst the direction of effect is unaltered, the uncertainty of assumptions, underlying our health model, can halve or significantly magnify estimates of the health and macroeconomic burdens of palm cooking oil in Thailand.

## Discussion

Our simulations illustrate the health-related economic, disease and population burden of palm cooking oil consumption in a high-consuming country context, Thailand. In calculating these burdens, it is acknowledged that substitution of palm oil with other dietary oils may not be possible in all processed food applications and some consumption of dietary oils is unavoidable. However, by focussing on the burden attributable to household consumption of palm cooking oil in Thailand, and employing a nutrient matching methodology, we isolate the ‘pure’ burden placed on the Thailand economy and its population from palm cooking oil consumption. As such, our analysis does not allow for the changes in consumption patterns that might result from policy instruments such as taxes, subsidies or regulation. Any consumption changes, outside the palm oil sector, result from the health cost and productivity-related 0.003% expansion in the Thai economy, only. These aggregate impacts are small. Differential impacts, exhibited at the household level, are driven, primarily, by relative differences in consumption patterns of palm cooking oil. However, the absence of distributional impacts across sectors, and across food sectors in particular, confirms that our estimates reflect the pure burdens relating to consumption of palm cooking oil in isolation, and not broader dietary change.

By using the same model framework to measure total MI and stroke burdens from all causes, we find that palm cooking oil burdens constitute approximately 0.5% of total MI and stroke burdens, the magnitude of which varied by ± 50% under our sensitivity analyses. Since our nutrient matching simulations assume no overall change in the total amount of dietary oils consumed, they reflect only the *additional* burden that, without changing diets, will result from consumption of palm cooking oil in Thailand over the next 20 years. Combined with the fact that our burden estimates also isolate household consumption of palm cooking oil from the use of palm oil in the processed food sector, our results indicate that health-related disease and economic burdens could be reduced by replacing consumption of palm cooking oil with other oils. However, the practicalities of removing the burden may result in other consequences if they result in substitution in the diet or require costly implementation.

Extrapolating from our results, it is reasonable to assume that any burden from palm cooking oil consumption in Thailand is likely to be present in a similar proportion in other high consuming nations, and consumption is known to be high in many countries in South and South East Asia. It is likely that the broader implications of palm oil policies on economic production and the environment will continue to dominate the global debate, but the implications of palm oil consumption on health may also warrant consideration and influence policy recommendations.

In terms of methodology, the integrated modelling approach, used to simulate these disease burdens, has the strength of combining a multi-sector CGE model, which comprehensively captures economic spill-overs, interactions and wage effects to robustly value productive labour, with other sub-models. The sectoral nature of the CGE approach allows the full integration of a health sub-model to capture transmission between consumption of food commodities and health outcomes, the outputs of which are, in turn, fed into a fully integrated demographic model. The resulting model not only provides an important valuation of economy-wide effects but also enables the decomposition of different aspects, such as the health and population burdens in a single general equilibrium application. However, the complex nature of the model means that detailed sectoral and household aggregation is data intensive and limited. This has resulted in aggregation of the processed food sector where, although some substitution of palm oil may be possible, the practicalities of reformulation and impact on palatability cannot be captured, and this has partially influenced our focus on household consumption of dietary palm cooking oil. The health effects we have captured relate changes in SFA, MUFA and PUFA consumption to MI and stroke via changes in the total-to-HDL cholesterol ratio, but additional exposures and health conditions, which may be of relevance, have been left for future consideration. Sensitivity analyses of the key health parameters resulted in ± 50% variations in disease burden impact for upper and lower bounds of the 95% confidence intervals. This demonstrates that the magnitudes of burden estimates are sensitive to health parameter assumptions, but provides a helpful estimate of the range of burden estimates. One further limitation of the results, presented, is that the ‘total disease burden’ scenario specification, in this application, does not produce the broader economic and environmental impacts, which are likely to result from changes in oil palm production patterns. These impacts could be captured if a policy instrument was employed in the simulation. Policy analysis is a strength of the CGE framework, and an example of reducing palm oil consumption using a sales tax has been published elsewhere [48]. However, whilst the substitution effects within an equilibrium model ensure realistic policy simulations, they contaminate estimation of the pure disease burden effect, which is the focus of this study. Similarly, likely changes in land use, provoked by substitution away from palm oil, could, for example, be an important consideration in this context, and could also be captured in a policy-focussed study [48].

## Conclusion

Our simulations indicate that consumption of palm cooking oil, rather than other dietary oils, are associated with negative health and economic burdens in Thailand, and, by extension, in high consuming countries. Since our analysis focuses on palm cooking oil, and assumes no overall reduction in dietary oil consumption, our burden estimates, which constitute 0.5% of the total burdens of MI and stroke, are an important indicator of the need for further assessment of the implications of palm oil consumption. Furthermore, we have demonstrated that development of integrated sectoral model frameworks is possible and such tools may be useful in assessing economic and health-related disease burdens. Further analyses, using similar tools, to assess the potential impacts of the use of palm-oil in other food applications and consideration of the implications of trade in palm cooking oil, would be highly relevant to inform policy makers.

## Additional file

Additional file 1: Appendix A: Sub models of the Integrated Framework. Appendix B: Additional Results. Figure S1: Decomposition of Household Income and Household Consumption. Table S1: Nutrient Matching Economic Results Decomposition. Table S2: Nutrient Matching Nutrition, Biomarker and Health Results Decomposition. Table S3: Nutrient Matching Demographic and workforce Results Decomposition (DOCX 43 kb)

## Abbreviations

AIDS: Almost Ideal Demand System
CGE: Computable general equilibrium
MI: Myocardial infarction
MUFA: Monounsaturated fatty acid
PUFA: Polyunsaturated fatty acid
SAM: Social accounting matrix
SFA: Saturated fatty acid
