# Supplementary material for: Evidence on the magnitude of the economic, health and population effects of palm cooking oil consumption: an integrated modelling approach with Thailand as a case study
Source: Popul Health Metr. 2019 Aug 16;17:12. doi: 10.1186/s12963-019-0191-y (PMC6697922; doi:10.1186/s12963-019-0191-y)
Supplement: Supplementary file 1 — Appendix A: Sub models of the Integrated Framework. Appendix B: Additional Results. Figure S1: Decomposition of Household Income and Household Consumption. Table S1: Nutrient Matching Economic Results Decomposition. Table S2: Nutrient Matching Nutrition, Biomarker and Health Results Decomposition. Table S3: Nutrient Matching Demographic and workforce Results Decomposition (DOCX 43 kb) [file 12963_2019_191_MOESM1_ESM.docx]

# **Appendices**

**Appendix A: Sub models of the Integrated Framework**

**Sub-models**

*Nutrition*

The link between the macroeconomic model, which captures household consumption of food (and non-food) commodities and health sub-model, which translates changes in total-to-HDL cholesterol to clinical outcomes was achieved by incorporating nutritional measurements in the model framework and converting them to total-to-HDL cholesterol changes. For each of the 9 regional households in the CGE model, data from the 2004-5 National Thai Food Consumption Survey (TFCS) [1,2]^[[1]](#footnote-1)^ and the 2011 Household Socio-Economic Survey (HSES) [3] were used in order to produce separate household- and commodity-specific coefficients related to total energy intake, and energy intakes from individual micronutrients including Saturated Fatty Acids (SFA), Monounsaturated Fatty Acids (MUFA), and Polyunsaturated Fatty Acids (PUFA). These household-specific average energy intake shares from SFA, MUFA and PUFA are used within the model to calculate average and stratified household-specific changes in the Total-to-HDL serum cholesterol ratio biomarker (ΔC) levels using the published equation from [4]:

ΔC_h,t_ = α^SFA^ SFA_h,t_ + α^MUFA^ MUFA_h,t_ + α^PUFA^ PUFA_h,t_ h ε H, t ε T

for each of our nine household types (H) and 20 time periods (T) and where the semi elasticities α^SFA^, α^MUFA^ and α^PUFA^ take the values 0.003, -0.026 and 0.032 respectively. These changes in SFA, MUFA and PUFA have been demonstrated to be correlated to changes in consumers’ serum cholesterol levels [4] (and are used as exposure inputs for the health model published by [5]).

*Health*

Our health model framework is primarily set up to model average serum cholesterol biomarker changes at the regional household level, however, by using individual-specific cholesterol level data this method was adjusted, allowing the computation of intra-household biomarker distributions from which clinical health outcomes were refined. The method employed used household-specific average changes in biomarker levels, and then shifted intra-household distributions by the mean according to the formula:

C^strata^_h,s,t_ = C^strata^_h,s,t-1_ + ΔC_h,t_ h ε H, s ε STRATA, t ε T

A set of 10 support points (STRATA) for each intra-household biomarker distribution were selected and 11 sets of lookup-tables for clinical health outcomes covering 10 intervals were stratified such that the 10 initial support points were midpoints of the clinical outcome intervals. Frequency distributions for the 10 intervals were produced from individual-level cholesterol data from the 2008-2009 Thailand National Health Examination Thai-HES Survey [6,7].^[[2]](#footnote-2)^

Data from the 2008-2009 Thai-HES Survey [6] was used to simulate age- and gender-specific lookup tables for clinical outcomes. Random sampling of 10,000 simulated individuals from normal distributions with means and standard deviations derived from the Thai-HES survey, was complemented by calculation of relative hazards for key events including non-fatal MI, non-fatal stroke, fatal MI, fatal stroke using an established empirical methodology [8] and relying on previously established log relative risks [9]. Individual risk was, subsequently, calculated from simulated alternative cause fatality rates, based on a competing risks algorithm [10], and from Global Burden of Disease data on rates of MI and stroke [11] and WHO data on all-cause mortality [12].

Relative hazards were stratified according to gender, rural/urban location, and 13 five-year age groups including 11 working age groups (15-64), and two retirement age groups ‘65-69’ and ‘70+’. Lookup tables were established for incidence and excess mortality of two illnesses, myocardial infarction (MI) and stroke, and the chosen biomarker support range ([2.0;7.0]) was chosen so as to encompass the full range of potential consumer-specific biomarker levels. Altogether, 11 sets of lookup tables were established, covering 10 equidistant intervals for the biomarker. Based on the simulated lookup tables, the clinical health outcome module was developed to encompass both a set of discontinuous spline-functions and a set of fitted 10^th^ degree polynomials – all gender, rural/urban, and age group-specific. For the current paper, the health module utilized fitted 10^th^ degree polynomials for calculation of gender, rural/urban, and age group-specific clinical health outcomes covering incidence and excess mortality rates for MI and stroke.

*Demographics*

The demographic module was designed to mirror the population stratification of the health and nutrition modules. A set of 2010-35 Thai regional population projections were obtained from the Office of the National Economic and Social Development Board [13,14]. The projections were age- and gender-specific (5 year age groups), and covered 8 regions which mapped one-to-one with our 5 region aggregation and allowed for rural/urban population splits of all regions excluding Bangkok. The aggregated data set was subsequently employed to calibrate our demographic model, encompassing a compact set of equations for calculating Deaths, Births, Migration, and Population levels. The demographic model is published in [15]. The 2010-35 population projection data set did not include information about underlying demographic parameter assumptions. Instead, age- and gender-specific parameters were obtained from the 2015 revision of UN population projections [16], and the demographic model calibration was completed through dynamic calibration of transition probabilities between 5 year age groups.

The clinical health outcome module, combined with the demographic module, produces numbers of MI and stroke incident cases and premature deaths. Patient incidence numbers are subsequently employed to calculate Years Lost due to Disability (YLD) patient morbidity impacts, informal caregiver time losses, and formal hospital costs, while the numbers of age-specific premature deaths are used to calculate patient-specific mortality-related changes to the workforce. Computation of YLD morbidity impacts for MI and stroke are based on YLD weights from the literature [17]^[[3]](#footnote-3)^, while calculations of caregiver time losses for stroke, including leisure and worktime losses, are based on Thai-specific average time loss estimates [18].^[[4]](#footnote-4)^ Thai-specific hospital unit costs were also derived from Thai-specific studies, including MI-related hospital unit costs [19] and stroke-related hospital unit costs [20], while workforce impacts were corrected for Thai-specific workforce participation rates [21]. 2015 base year hospital unit costs were calculated from 2005 MI/2008 Stroke baseline hospital unit costs and general 2005-2015 Thai GDP inflation rates, while increases in hospital unit costs over our 2016-2035 time horizon were calculated from general price changes in our market for healthcare services.

**Appendix B: Additional Results**

|  |  |
| --- | --- |

Figure S1: Decomposition of Household Income and Household Consumption

**Additional Results for Nutrient Matching Scenario**

**Table S1: Nutrient Matching Economic Results Decomposition**

| **economic** |  |  |
| --- | --- | --- |
| ***Real GDP & sales tax*** |  |  |
|  | mn USD | % of GDP |
| Δreal GDP | 308 | 0.003% |
|  |  |  |
|  |  |  |
| ***Real Consumption/welfare*** |  |  |
|  | mn USD | % of rHC |
| Δreal Household Consumption (rHC) | 152 | 0.0028% |
| - Bangkok | 34 | 0.0033% |
| - Central region (exc Bangkok) | 30 | 0.0019% |
| - North region | 34 | 0.0043% |
| - Northeast region | 23 | 0.0018% |
| - South region | 31 | 0.0035% |
| ***Health system costs*** |  |  |
|  | mn USD | % of GDP |
| Δreal Health System Expenses |  |  |
| - formal hospital | -147 | -0.45% |
| - myocardial infarction | -145 | -0.47% |
| - stroke | -3 | -0.13% |

**Table S2: Nutrient Matching Nutrition, Biomarker and Health Results Decomposition**

| **nutrition, biomarker, health** |  |  |
| --- | --- | --- |
| ***Nutrition (long run indicators)*** |  |  |
|  | %-points | % of share |
| ΔSFA energy intake share (long run, 2035) | -0.618% | -6.494% |
| ΔMUFA energy intake share (long run, 2035) | -0.228% | -2.997% |
| ΔPUFA energy intake share (long run, 2035) | 0.742% | 13.670% |
| ***Biomarker (cumulative indicators)*** |  |  |
|  | cum. chg. | % of total |
| ΔTotal-to-HDL cholesterol ratio | -0.080 | -1.74% |
| ***Health (cumulative indicators)*** |  |  |
|  | cases | % of total |
| ΔPatient Incident Cases | - 10,919 | -0.29% |
| - myocardial infarction | -8,280 | -0.49% |
| - stroke | -2,639 | -0.13% |
| ΔPatient premature deaths | -5,577 | -0.29% |
| - myocardial infarction | -4,683 | -0.46% |
| - stroke | -894 | -0.10% |
|  | pers-yrs | % of total |
| ΔPatient Disease Burden (YLD) | -2,211 | -0.12% |
| - myocardial infarction | -13 | -0.49% |
| - stroke | -2,197 | -0.12% |
| ΔPatient Worktime Loss | -1,037 | -0.17% |
| - myocardial infarction | -6 | -0.56% |
| - stroke | -1,031 | -0.17% |
| ΔCaregiver Time Loss | -4,786 | -0.13% |
| - stroke | -4,786 | -0.13% |
| - work time | -1,785 | -0.12% |
| - leisure time | -3,001 | -0.13% |

**Table S3: Nutrient Matching Demographic and workforce Results Decomposition**

| **Demographic and workforce** | | |
| --- | --- | --- |
| ***Demographic*** |  |  |
|  | #pers-yrs | % of total |
| Δpopulation (cumulative) | 49,935 | 0.0037% |
| - Bangkok^1^ | 9,282 | 0.0056% |
| - Central region (exc Bangkok)^1^ | 12,915 | 0.0032% |
| - North region^1^ | 8,838 | 0.0040% |
| - Northeast region^1^ | 9,071 | 0.0025% |
| - South region^1^ | 9,829 | 0.0052% |
| - urban^1^ | 40,293 | 0.0053% |
| - rural^1^ | 9,642 | 0.0017% |
|  | #persons | % of total |
| Δpopulation (long run, 2035) | 5,004 | 0.0072% |
| - Bangkok^1^ | 880 | 0.0108% |
| - Central region (exc Bangkok)^1^ | 1,309 | 0.0059% |
| - North region^1^ | 899 | 0.0080% |
| - Northeast region^1^ | 932 | 0.0051% |
| - South region^1^ | 985 | 0.0099% |
| - urban^1^ | 4,257 | 0.0094% |
| - rural^1^ | 747 | 0.0031% |
| ***Workforce*** |  |  |
|  | #persons | % of total |
| Δworkforce (cumulative) | 15,364 | 0.00% |
| - urban^1^ | 12,139 | 0.00% |
| - rural^1^ | 3,224 | 0.00% |
|  | #persons | % of total |
| Δworkforce (long run, 2035) | 1,299 | 0.00% |
| - urban^1^ | 1,090 | 0.01% |
| - rural^1^ | 209 | 0.00% |

**References for Appendices**

[1] Kosulwat V, Rojroongwasinkul N, Boonpraderm A. Food consumption data of Thailand (in Thai). Bangkok. National Bureau of Agricultural Commodity and Food Standards, Ministry of Agriculture and Cooperatives. 2006.

[2] Jitnarin N, Kosulwat V, Rojroongwasinkul N, Boonpraderm A, Haddock CK, Poston WSC. Risk Factors for Overweight and Obesity among Thai Adults: Results of the National Thai Food Consumption Survey. Nutrition. 2010;2(1):60-74.

[3] NSO. 2011 Household Socio-Economic Survey (Electronic data). National Statistical Office Thailand. Bangkok. 2014.

[4] Mensink RP, Zock PL, Kester AD, Katan MB. Effects of dietary fatty acids and carbohydrates on the ratio of serum total-to-HDL cholesterol and on serum lipids and apolipoproteins: a meta-analysis of 60 controlled trials. Am J Clin Nutr. 2003;77:1146 55.

[5] Basu S, Babiarz KS, Ebrahim S, Vellakkal S, Stuckler D, Goldhaber-Fiebert JD. Palm oil taxes and cardiovascular disease mortality in India: economic-epidemiologic model. BMJ. 2013;347:f6048

[6] NHESO. Thailand National Health and Examination Survey 2008-2009. National Health Examination Survey Office. Health Systems Research Institute. Thailand. 2009.

[7] Aekplakorn W, Chariyalertsak S, Kessomboon P, Sangthong R, Inthawong R, Putwatana P. Prevalence and Management of Diabetes and Metabolic Risk Factors in Thai Adults. Diabetes Care 2011;34(9):1980-5.

[8] Lim SS, Gaziano TA, Gakidou E, Reddy KS, Farzadfar F, Lozano R. Prevention of cardiovascular disease in high-risk individuals in low-income and middle-income countries: health effects and costs. Lancet 2007;370(9604):2054-62.

[9] Lewington S, Whitlock G, Clarke R, Sherliker P, Emberson J, Halsey J. Blood cholesterol and vascular mortality by age, sex, and blood pressure: a meta-analysis of individual data from 61 prospective studies with 55 000 vascular deaths. Lancet 2007;370(9604):1829-39.

[10] Prentice RL, Kalbfleisch JD, Peterson Jr JD, Flournoy N, Farewelland VT, Breslow NE. The Analysis of Failure Times in the Presence of Competing Risks. Biometrics 1978;34(4):541-54

[11] Lozano R, Naghavi M, Foreman K, Lim S, Shibuya K, Aboyans V, et al. Global and regional mortality from 235 causes of death for 20 age groups in 1990 and 2010: a systematic analysis for the Global Burden of Disease Study 2010. Lancet 2012;380(9859):2095-2128.

[12] WHO. World Health Organization Mortality Database. World Health Organization. Geneva. 2014. <http://www.who.int/healthinfo/mortality_data/en/>

[13] NESDB. Population projection for Thailand 2010-2040. Office of the National Economic and Social Development Board. Bangkok. 2013a.

[14] NESDB. Population projections at regional and municipality level 2010-2035. Electronic data. Office of the National Economic and Social Development Board. Bangkok. 2013b.

[15] Jensen H, Keogh-Brown MR, Shankar B, Basu S, Cuevas S, Dangour AD. Palm oil and dietary change: Application of an integrated macroeconomic, environmental, demographic, and health modelling framework for Thailand. Food Policy (In revision)

[16] UN. World Population Prospects, the 2015 Revision (Electronic Data). United Nations. URL: <http://esa.un.org/unpd/wpp/index.htm>. 2015. (accessed 14. September 2015)

[17] WHO. WHO methods and data sources for global burden of disease estimates 2001-2011. Global Health Estimates Technical Paper WHO/HIS/HSI/GHE/2013.4. World Health Organization. Geneva. 2013

[18] Riewpaiboon A, Riewpaiboon W, Ponssongnern K, van den Berg B. Economic valuation of informal care in Asia: A case study of care for disabled stroke survivors in Thailand. Soc Sci Med. 2009.;69(4):648-53.

[19] Anukoolsawat P, Sritara P, Teerawattananon Y. Costs of Lifetime Treatment of Acute Coronary Syndrome at Ramathibodi Hospital. Thai Heart J. 2006;19(4):132-143.

[20] Khiaocharoen O, Pannarunothai S, Zungsontiporn C. Cost of acute and sub-acute care for stroke patients. J Med Assoc Thai. 2012;95(10):1266-77.

[21] NSO. Key statistics of Thailand 2003-2007. National Statistical Office Thailand. Bangkok. 2008.

1. The 2004-5 TFCS data set was conducted in 2003-2005 by Institute of Nutrition, Mahidol University with support from the National Bureau of Agricultural Commodity and Food Standards, Thailand. TFCS data extracts were kindly supplied by Dr. Nipa Rojroongwasinkul, Institute of Nutrition, Mahidol University. [↑](#footnote-ref-1)
2. A few initial Total-to-HDL ratio biomarker outliers were eliminated from the database. [↑](#footnote-ref-2)
3. The severity of stroke differs strongly between cases. This is evidenced by the distribution of Thai stroke cases over four Barthel index-based disability categories [18]. In order to capture the distribution of case severity, the four Barthel-categories of the latter paper (all defined by Barthel index scores<95) were mapped to the four least severe categories (out of five) of 2010 Global Burden of Disease YLD disability weights for stroke [17]. Together, the two distributions of Thai-specific case severity shares and severity-specific YLD disease burdens were used to compute weighted Thai YLD disability weights for stroke (to be used for our patient disease burden calculations). In terms of MI, the two categories of 2010 Global Burden of Disease YLD disability weights were defined for stage 1 (first 2 days, most severe) and stage 2 (following 26 days, much less severe). For MI, we therefore decided to compute a weighted YLD disability weight using the relative duration of the two stages as weights. [↑](#footnote-ref-3)
4. Caregiver time losses for myocardial infarction (MI) were considered small due to short illness duration, and therefore not included in the study. E.g. the 2010 GBD study only attributes MI disease burden to the first 28 days of illness [17]. [↑](#footnote-ref-4)
